# Multimorbidity patterns in relation to polypharmacy and dosage frequency: a nationwide, cross-sectional study in a Japanese population

**DOI:** 10.1038/s41598-018-21917-6

**Published:** 2018-02-28

**Authors:** Takuya Aoki, Yosuke Yamamoto, Tatsuyoshi Ikenoue, Yoshihiro Onishi, Shunichi Fukuhara

**Affiliations:** 1 Department of Healthcare Epidemiology, School of Public Health in the Graduate School of Medicine, Kyoto University, Kyoto, Japan; 2 Institute for Health Outcomes and Process Evaluation Research (iHope International), Kyoto, Japan; 3 Department of General Medicine, Shirakawa Satellite for Teaching And Research (STAR), Fukushima Medical University, Fukushima, Japan; 4 Center for Innovative Research for Communities and Clinical Excellence (CIRC2LE), Fukushima Medical University, Fukushima, Japan

## Abstract

In the present study, we aimed to identify multimorbidity patterns in a Japanese population and investigate whether these patterns have differing effects on polypharmacy and dosage frequency. Data was collected on 17 chronic health conditions via nationwide cross-sectional survey of 3,256 adult Japanese residents. Factor analysis was performed to identify multimorbidity patterns, and associations were determined with excessive polypharmacy [concurrent use of ≥ 10 prescription or over-the-counter (OTC) medications] and higher dosage frequency ( ≥ 3 doses per day). Secondary outcomes were the number of concurrent prescription medications and the number of concurrent OTC medications. We used a generalized linear model to adjust for individual sociodemographic characteristics. Five multimorbidity patterns were identified: cardiovascular/renal/metabolic, neuropsychiatric, skeletal/articular/digestive, respiratory/dermal, and malignant/digestive/urologic. Among these patterns, malignant/digestive/urologic and cardiovascular/renal/metabolic patterns showed the strongest associations with excessive polypharmacy and the number of concurrent OTC medications. Malignant/digestive/urologic, respiratory/dermal, and skeletal/articular/digestive patterns were also associated with higher dosage frequency. Multimorbidity patterns have differing effects on excessive polypharmacy and dosage frequency. Malignant/digestive/urologic pattern may be at higher risk of impaired medication safety and increased treatment burden, than other patterns. Continued study is warranted to determine how to incorporate multimorbidity patterns into risk assessments of polypharmacy and overall treatment burden.

## Introduction

Multimorbidity, which is defined as the co-occurrence of multiple chronic or acute diseases and medical conditions in an individual, is increasingly becoming a major concern in primary care^1^. Multimorbidity patterns are now the focus of researchers who aim to provide a better understanding of the complex nature of multimorbid health conditions. In recent studies, statistical approaches such as factor analysis were used to identify the nonrandom cluster patterns of individual health conditions into groups of multimorbid conditions^2–5^. While there is a growing body of evidence showing multimorbidity patterns in various populations, only a few studies have been conducted to investigate the associations of these patterns with clinical outcomes^6,7^.

The pharmacomanagement of multimorbidity often requires polypharmacy, which is defined as the use of multiple medications concurrently for the treatment of one to several medical conditions, and high dosage frequency. Polypharmacy and high dosage frequency are both associated with high rates of adverse drug reactions, poor adherence, and hospitalization^8–10^. In addition, polypharmacy and high dosage frequency induce treatment burden, which is defined as the “work” of being a patient and its effect on the patient’s quality of life^11^. Although a number of studies have investigated the associations of one-dimensional index of multimorbidity (i.e., the total number of chronic health conditions) with polypharmacy and dosage frequency^12,13^, the question of whether nonrandom cluster patterns of chronic health conditions have differing effects on these outcomes remains unanswered. Such a one-dimensional index may be too crude to fully elucidate the effects of multimorbidity on medication usage in real world practice. Cluster patterns of health conditions may have a synergistic effect on the number of medications, and specific patterns may be related to an increase in dosage frequency.

In the present study, we aimed to identify multimorbidity patterns in a Japanese population, and determine the extent to which multimorbidity patterns have differing effects on polypharmacy and dosage frequency.

## Methods

### Setting and Participants

The data used for this study were collected from the Norm Study conducted in 2016. The Norm Study was a nationwide cross-sectional survey to collect data on health-rated quality of life, health conditions, healthcare utilization, and sociodemographic characteristics in a Japanese general population. A quota sampling method was used to select representative samples of the Japanese general population, aged 16–84 years, from a residents’ panel administered by the Nippon Research Center. This large panel is composed of approximately 300,000 residents in Japan. In this study, we set quotas with regard to age, sex, and residential area to make our sample representative of the demographic distribution of Japan as shown in the most recent census data. Data collection was either Web-based for patients aged ≤69 years or mail-based for those aged ≥70 years. In total, 3,307 participants completed the questionnaire. Analysis was restricted to data collected from adult residents aged ≥18 years, with complete morbidity data.

### Measures

#### Morbidity Status

A structured questionnaire, used in previous studies, was chosen to collect data on a wide range of chronic conditions to assess individual morbidity status^14^. Previous studies have shown that assessment of morbidity using self-reported data is able to predict clinical outcomes comparably with measures based on administrative data^15,16^. To determine the occurrence of chronic health conditions, respondents were asked the question “Has a doctor/nurse/paramedic ever told you that you had following chronic health conditions?” with 17 response options: hypertension, diabetes, dyslipidemia, stroke, cardiac diseases (e.g., coronary heart disease, heart failure, arrhythmia), chronic respiratory diseases (e.g., asthma, chronic obstructive pulmonary disease), digestive diseases (e.g., gastroesophageal reflux disease, cirrhosis), kidney diseases (e.g., chronic kidney disease), urologic diseases (e.g., prostatic hypertrophy, overactive bladder), arthritis or rheumatism (e.g., osteoarthritis, rheumatoid arthritis), lumbar diseases (e.g., lumbar spinal stenosis, osteoporosis), neurologic diseases (e.g., epilepsy, dementia), mental disorders (e.g., depression), endocrine diseases (e.g., thyroid disorders), malignancy, vision abnormalities, or skin diseases (e.g., atopic dermatitis). Participants responded to each option on binary scale (“yes” or “no”). Multimorbidity was defined as the occurrence of ≥2 chronic health conditions^17^.

#### Polypharmacy and Dosage Frequency

The primary outcome measures in this study were excessive polypharmacy and higher dosage frequency. Excessive polypharmacy was defined as the concurrent use of ≥10 prescription or over-the-counter (OTC) medications, a level previously associated with increased hospital admissions and a decline in nutritional status, functional ability, and cognitive capacity in patients with multimorbidity^18,19^. Excessive polypharmacy was assessed using a structured questionnaire. Patients were asked to provide a count of the medications that were chronically prescribed to them, along with the number of OTC medications. Externally applied medications were excluded from this assessment. Dosage frequency was also assessed by self-administered questionnaire. Dosage frequency was divided into two categories: <3 doses per day (lower dosage frequency) and ≥3 doses per day (higher dosage frequency), in accordance with the results of a systematic review that found lower levels of adherence in patients who took medications ≥3 times per day, as compared to once daily^20^. Secondary outcome measures were the number of concurrent prescription medications and the number of concurrent OTC medications.

#### Covariates

Covariates were selected on the basis of evidence from previous studies that suggested confounding relationships between multimorbidity patterns and the outcomes explored in this study^21,22^. We included covariates for age, sex, years of education, and household income. All covariates were evaluated as categorical variables by a self-administered questionnaire.

### Statistical analysis

A two-step procedure was applied to determine the extent to which multimorbidity patterns vary in their associations with polypharmacy and dosage frequency.

#### Factor Analysis

Multimorbidity patterns were determined using an exploratory factor analysis of participants with complete morbidity data. We applied multidimensional item response theory and promax rotation for accurate estimation, since chronic health conditions were coded as dichotomous variables^23^. Local independence of items is an important assumption of the multidimensional IRT model. In this study, since the survey items were a wide range of chronic health conditions, we considered that there was no dependency between items and that theoretically local independence could be assumed. Participants without multimorbidity or those having no disease were included to prevent the overestimation of correlations between diagnosis groups, which may have biased the resulting correlation matrix^24^. For this reason we performed factor analysis using data from all adult participants. The optimal number of factors was determined based on a parallel analysis^25^. A factor loading greater than 0.30 was considered meaningful and used as criteria for item selection. Multimorbidity factor scores for each individual participant were derived using a maximum a posteriori (MAP) estimation, incorporating both factor loadings and category threshold^23^. For ease of interpretation, we categorized scores into quartiles, because no known cut-off points are available.

#### Associations of multimorbidity patterns with outcomes

Data were analyzed from adult outpatients who had regularly visited ≥1 medical institution, and who were at risk of polypharmacy. Multivariable logistic regression analyses included the following possible confounding variables as covariates: age, sex, years of education, and annual household income. Poisson regression analysis was used to investigate associations between multimorbidity factor scores and the number of concurrent prescription medications and the number of OTC medications, separately. For each analysis, we used a two-sided significance level of *P* = 0.05. Missing data for independent and dependent variables were accounted for using multiple imputation, using a fully conditional specification. Statistical analyses were conducted using R version 3.3.2 (R Foundation for Statistical Computing, Vienna, Austria; www.R-project.org/) and psych, mirt, and mice packages for statistical analyses.

### Ethics

The institutional review board of the Institute for Health Outcomes and Process Evaluation Research (iHope International) provided ethical approval for this study. All methods were performed in accordance with the guidelines and regulations of the institutional review board of the iHope International. A written informed consent was obtained from every participant.

## Results

Out of a total of 3,307 study participants, 3,256 were aged ≥18 years and had complete data for all chronic health conditions, and were thus included in the factor analysis. The overall prevalence of multimorbidity was 29.9%. For older participants, aged ≥65 years, the prevalence of multimorbidity was 62.8%. A total of 1,480 (45.5%) participants were regular outpatients, having visited ≥1 medical institution. Table 1 shows the characteristics of the study population, as well as those of outpatients. Excessive polypharmacy was found in 101 patients (6.8%), and higher dosage frequency was found in 351 patients (23.7%) among outpatients.

**Table 1:** Participants’ characteristics: N (%).

Characteristic	Total participants (N = 3,256)	Outpatients^*^ (N = 1,480)
Gender		
Male	1617 (49.7)	752 (50.8)
Female	1639 (50.3)	728 (49.2)
Data missing	0	0
Age (year)		
18–29	525 (16.1)	135 (9.1)
30–44	840 (25.8)	259 (17.5)
45–64	1092 (33.5)	499 (33.7)
65–74	514 (15.8)	357 (24.1)
≧ 75	285 (8.8)	230 (15.5)
Data missing	0	0
Education level		
Less than high school	107 (3.3)	67 (4.5)
High school	1249 (38.3)	588 (39.7)
Junior college	337 (10.4)	142 (9.6)
More than or equal to college	1423 (43.7)	571 (38.6)
Data missing	140	112
Annual household income (million JPY)		
<3.00 (≒27,000 US dollar)	810 (24.9)	402 (27.2)
3.00–4.99	934 (28.7)	443 (29.9)
5.00–6.99	621 (19.1)	250 (16.9)
7.00–9.99	503 (15.4)	210 (14.2)
≧ 10.00	346 (10.7)	143 (9.7)
Data missing	42	32
Number of morbidities		
0	1468 (45.1)	245 (16.6)
1	815 (25.0)	461 (31.1)
≧ 2	973 (29.9)	774 (52.3)
Number of concurrent medications^†^		
0–4	2712 (83.3)	968 (65.4)
5–9	405 (12.4)	401 (27.1)
≧10	101 (3.1)	101 (6.8)
Data missing	38	10
Number of doses per day		
0–2	2895 (88.9)	1119 (75.6)
≧ 3	351 (10.8)	351 (23.7)
Data missing	10	10

^*^Adult outpatients regularly visiting ≥1 medical institution.

^†^Prescription and over-the-counter medications.

**Figure 1:**
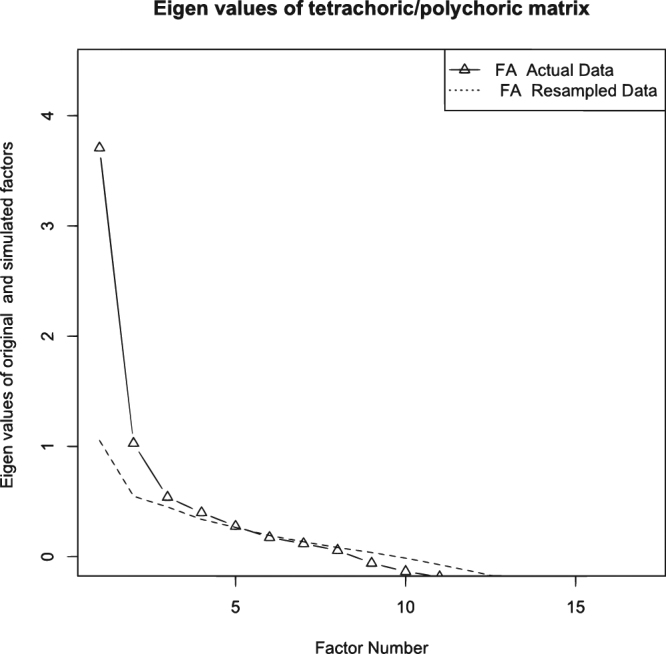
Scree plots of the actual data and the resampled data for 17 chronic health conditions

Figure 1 shows scree plots of both actual data and resampled data for 17 chronic health conditions. Parallel analysis suggested a five-factor solution, where eigenvalues of the actual data were larger than those derived from resampled data when the number of factors was five or less.

Table 2 shows factor loadings for the five-factor solution following an exploratory factor analysis, based on multidimensional item response theory (loadings exceeding the cut-off of 0.30 appear in bold). The following factors were identified and labeled:

**Table 2:** Factor loadings for the five-factor solution following an exploratory factor analysis in a general adult population^*^ (N = 3,256).

	Factor1	Factor2	Factor3	Factor4	Factor5
Hypertension	**0**.**76**	−0.04	0.13	−0.12	−0.01
Diabetes	**0**.**74**	−0.07	−0.24	0.13	0.05
Dyslipidemia	**0**.**58**	−0.06	0.18	0.04	−0.14
Stroke	**0**.**61**	0.18	0.08	−0.06	0.03
Cardiac diseases	**0**.**40**	−0.21	0.11	−0.02	0.19
Chronic respiratory diseases	0.02	0.06	0.01	**0**.**68**	0.02
Digestive diseases	0.05	0.18	**0**.**45**	0.09	**0**.**30**
Kidney diseases	**0**.**37**	0.10	0.12	0.13	0.29
Urologic diseases	−0.03	−0.02	−0.02	−0.01	**0**.**97**
Arthritis & rheumatism	0.08	−0.34	**0**.**46**	0.10	−0.01
Lumbar diseases	0.03	0.01	**0**.**81**	0.03	0.01
Neurologic diseases	0.19	**0**.**36**	0.18	0.21	0.14
Mental disorders	−0.01	**0**.**60**	0.19	0.24	0.00
Endocrine diseases	0.25	−0.31	−0.03	−0.10	0.15
Malignancy	0.08	−0.01	0.08	−0.02	**0**.**45**
Vision abnormalities	0.16	−0.58	0.26	0.23	0.13
Skin diseases	−0.10	−0.05	−0.02	**0**.**62**	−0.01

^*^By multidimensional item response theory and promax rotation.

Loadings are bolded if they exceed 0.30.

Factor 1: cardiovascular/renal/metabolic diseases (hypertension, diabetes, dyslipidemia, stroke, cardiac diseases, and kidney diseases)

Factor 2: neuropsychiatric diseases (mental disorders and neurologic diseases)

Factor 3: skeletal/articular/digestive diseases (arthritis or rheumatism, lumbar diseases, and digestive diseases)

Factor 4: respiratory/dermal diseases (chronic respiratory diseases and skin diseases)

Factor 5: malignant/digestive/urologic diseases (malignancy, digestive, and urologic diseases)

**Figure 2:**
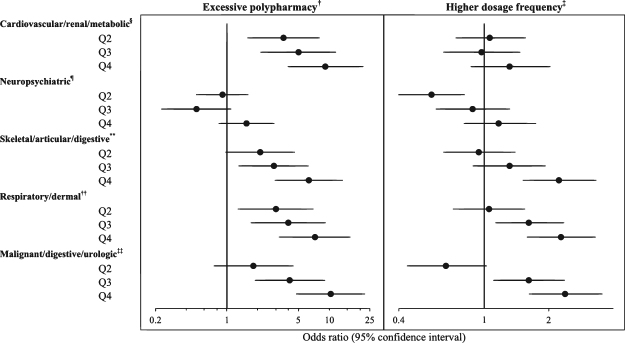
Associations of multimorbidity factor scores with excessive polypharmacy and higher dosage frequency in adult outpatients (N = 1,480)^*^. ^*^Adjusted for age, sex, education level, annual household income; Each factor score was included individually in the model; Reference, Q1. ^†^Use of ≥10 concurrent medications. ^‡^ ≥ 3 doses per day. ^§^Cardiovascular/renal/metabolic factor score quartiles: Q1, −0.30 to −0.15; Q2, −0.09 to 0.62; Q3, 0.62 to 1.21; Q4, 1.21 to 3.09. Neuropsychiatric factor score quartiles: Q1, −1.81 to −0.40; Q2, −0.40 to −0.14; Q3, −0.13 to 0.05; Q4, 0.06 to 1.41. ^**^Skeletal/articular/digestive factor score quartiles: Q1, −2.82 to −0.98; Q2, −0.97 to −0.37; Q3, −0.37 to −0.11; Q4, −0.11 to 0.22. ^††^Respiratory/dermal factor score quartiles: Q1, −0,22 to −0.12; Q2, −0.07 to 0.29; Q3, 0.31 to 0.73; Q4, 0.74 to 2.81. ^‡‡^Malignant/digestive/urologic factor score quartiles: Q1, −3.41 to −0.69; Q2, −0.69 to −0.26; Q3, −0.26 to −0.04; Q4, −0.03 to 0.15.

Figure 2 shows adjusted associations between multimorbidity factor scores and primary outcomes. Malignant/digestive/urologic and cardiovascular/renal/metabolic scores had the strongest association with excessive polypharmacy [adjusted odds ratio (aOR) = 10.30, 95% confidence interval (CI): 4.78 to 22.20 for the malignant/digestive/urologic score highest quartile; 9.22, 95%CI: 3.96 to 21.45 for the cardiovascular/renal/metabolic score highest quartile, compared with the lowest quartile], followed by respiratory/dermal and skeletal/articular/digestive. In addition, malignant/digestive/urologic, respiratory/dermal, and skeletal/articular/digestive scores were also significantly associated with higher dosage frequency (aOR = 2.39, 95%CI: 1.62 to 3.54 for the malignant/digestive/urologic score highest quartile; 2.29, 95%CI: 1.59 to 3.29 for the respiratory/dermal score highest quartile; 2.24, 95%CI: 1.52 to 3.31 for the skeletal/articular/digestive score highest quartile, compared with the lowest quartile).

Table 3 shows the associations between multimorbidity factor scores and the number of concurrent medications. Cardiovascular/renal/metabolic, skeletal/articular/digestive, respiratory/dermal, and malignant/digestive/urologic scores were all associated with the number of both concurrent prescription medications and OTC medications. Of these, cardiovascular/renal/metabolic and malignant/digestive/urologic scores showed the strongest associations with the number of concurrent OTC medications.

**Table 3:** Associations between multimorbidity factor scores and the number of concurrent medications in adult outpatients (N = 1,480)^*^.

Multimorbidity factor score	Adjusted RR (95% CI)	
	Prescription medications	Over-the-counter medications
Cardiovascular/renal/metabolic^†^		
Q1 (lowest)	Reference	
Q2	1.17 (1.07 to 1.29)	1.54 (1.31 to 1.81)
Q3	1.24 (1.19 to 1.37)	1.68 (1.41 to 2.01)
Q4 (highest)	1.79 (1.62 to 1.98)	1.78 (1.47 to 2.15)
Neuropsychiatric^‡^		
Q1 (lowest)	Reference	
Q2	0.81 (0.75 to 0.87)	1.27 (1.08 to 1.48)
Q3	0.73 (0.66 to 0.80)	0.95 (0.79 to 1.14)
Q4 (highest)	1.01 (0.93 to 1.11)	1.11 (0.93 to 1.33)
Skeletal/articular/digestive^§^		
Q1 (lowest)	Reference	
Q2	1.10 (1.00 to 1.21)	1.33 (1.14 to 1.55)
Q3	1.41 (1.28 to 1.54)	1.30 (1.10 to 1.53)
Q4 (highest)	1.68 (1.53 to 1.85)	1.34 (1.12 to 1.59)
Respiratory/dermal		
Q1 (lowest)	Reference	
Q2	1.42 (1.29 to 1.55)	1.27 (1.09 to 1.48)
Q3	1.49 (1.36 to 1.63)	1.19 (1.02 to 1.40)
Q4 (highest)	1.87 (1.71 to 2.05)	1.30 (1.10 to 1.52)
Malignant/digestive/urologic^**^		
Q1 (lowest)	Reference	
Q2	1.11 (1.01 to 1.22)	1.40 (1.19 to 1.65)
Q3	1.46 (1.33 to 1.60)	1.62 (1.37 to 1.91)
Q4 (highest)	1.85 (1.68 to 2.03)	1.71 (1.44 to 2.04)

RR, risk ratio; CI, confidence interval.

^*^Adjusted for age, sex, education level, annual household income; Each factor score was included individually in the model.

^†^Cardiovascular/renal/metabolic factor score quartiles: Q1, −0.30 to −0.15; Q2, −0.09 to 0.62; Q3, 0.62 to 1.21; Q4, 1.21 to 3.09.

^‡^Neuropsychiatric factor score quartiles: Q1, −1.81 to −0.40; Q2, −0.40 to −0.14; Q3, −0.13 to 0.05; Q4, 0.06 to 1.41.

^§^Skeletal/articular/digestive factor score quartiles: Q1, −2.82 to −0.98; Q2, −0.97 to −0.37; Q3, −0.37 to −0.11; Q4, −0.11 to 0.22.

Respiratory/dermal factor score quartiles: Q1, −0,22 to −0.12; Q2, −0.07 to 0.29; Q3, 0.31 to 0.73; Q4, 0.74 to 2.81.

^**^Malignant/digestive/urologic factor score quartiles: Q1, −3.41 to −0.69; Q2, −0.69 to −0.26; Q3, −0.26 to −0.04; Q4, −0.03 to 0.15.

## Discussion

In a representative sample of Japanese adults, aged 18–84 years, our study identified five multimorbidity patterns with differing associations with both excessive polypharmacy and dosage frequency in outpatients. Malignant/digestive/urologic and cardiovascular/renal/metabolic patterns had the strongest association with excessive polypharmacy, and these results could be explained by the increase in OTC medications. Furthermore, malignant/digestive/urologic, respiratory/dermal, and skeletal/articular/digestive patterns were also associated with higher dosage frequency, all of which affect treatment burden as well as polypharmacy.

The results of this study concur with those of the systematic review, which demonstrated similar relationships for three multimorbidity patterns: cardiovascular and metabolic diseases, mental health problems, and musculoskeletal disorders^14^. Similarly, our study identified cardiovascular/renal/metabolic diseases, neuropsychiatric diseases, and skeletal/articular/digestive diseases. Comorbidity of chronic respiratory diseases, such as asthma, and common skin diseases, such as atopic dermatitis, is well-established^26^. In addition, we identified a malignancy pattern. Previously, high prevalence of comorbid chronic diseases has been reported among cancer survivors^27^, however, it has been unclear which chronic health conditions form a group with malignancy. The grouping of malignancy with digestive and urologic diseases in our study may be a consequence of the late effects of cancer and its treatment, because digestive (e.g., colon) and urologic (e.g., prostate) organs are relatively frequent as sites of primary lesions in cancer survivors^27^.

Previous studies have established associations of one-dimensional index of multimorbidity with the number of medications and the number of doses^12,13^. The present study adds to this body of evidence by comparing the potential effects of various multimorbidity patterns on polypharmacy and higher dosage frequency. In our study, malignant/digestive/urologic and cardiovascular/renal/metabolic patterns showed the strongest associations with excessive polypharmacy. In concordance with these findings, previous studies have suggested that polypharmacy and OTC medication use are common in patients with cancer or cardiovascular disease^28–30^. The malignant/digestive/urologic pattern was also associated with dosage frequency, suggesting that cancer survivors with multimorbid conditions may be at higher risk of impaired medication safety, and increased treatment burden, than other patterns.

To the best of our knowledge, this is the first study to compare the effects of different multimorbidity patterns on polypharmacy and dosage frequency. A key advantage of our study was its use of data from a nationwide representative sample of the Japanese general adult population, which allows for generalization of its results to the wider population. Furthermore, in addition to data on the use of prescribed medications, our study also included data on OTC medication usage, providing a more comprehensive assessment of polypharmacy. For accurate estimation in the case of dichotomous variables, we applied improved methods of identifying multimorbidity patterns and factor score calculation by using parallel analysis, multidimensional item response theory, and MAP estimation.

Our study had several potential limitations. First, since it relies on cross-sectional data, a causal association cannot be confirmed. Second, while the quota sampling method ensures that the sample is representative of the quota-defining characteristics, other characteristics might be disproportionately represented in the sample group. Therefore, some selection bias may have affected our results. Third, the use of self-reported data may have underestimated the prevalence of chronic disease in this population through misclassification bias. In addition, the measure of multimorbidity was only concerned with the number of chronic health conditions and did not take into consideration the severity of disease. Fourth, although self-reported health conditions are commonly used to identify multimorbidity patterns in a general population^2,4,5^, this method of assessment may have introduced selection bias because the residents’ panel did not include patients with diseases, such as advanced dementia. In addition, our participants were limited to residents with complete morbidity data. However, in our study, only 11 residents were excluded for incomplete morbidity data, and there was no statistical difference in the characteristics between the residents with complete morbidity data and the residents without complete morbidity data. Fifth, data on the types of medications taken were not collected, thus we could not evaluate the appropriateness of medications. However, polypharmacy is known as an important risk factor for potentially inappropriate medications^31^.

In conclusion, our study found that multimorbidity patterns have differing effects on both excessive polypharmacy and dosage frequency. Malignant/digestive/urologic pattern may be at higher risk of impaired medication safety and increased treatment burden, than other patterns. Continued study is warranted to determine how to incorporate multimorbidity patterns into risk assessments of polypharmacy and overall treatment burden.

### Data availability

The datasets generated and analyzed during the current study are available from the corresponding author on reasonable request.
